# A *tamB* homolog is involved in maintenance of cell envelope integrity and stress resistance of *Deinococcus radiodurans*

**DOI:** 10.1038/srep45929

**Published:** 2017-04-06

**Authors:** Jiangliu Yu, Tao Li, Shang Dai, Yulan Weng, Jiulong Li, Qinghao Li, Hong Xu, Yuejin Hua, Bing Tian

**Affiliations:** 1 Key Laboratory for Nuclear-Agricultural Sciences of Chinese Ministry of Agriculture and Zhejiang Province, Institute of Nuclear-Agricultural Sciences, Zhejiang University, Hangzhou, China

## Abstract

The translocation and assembly module (TAM) in bacteria consists of TamA and TamB that form a complex to control the transport and secretion of outer membrane proteins. Herein, we demonstrated that the DR_1462-DR_1461-DR_1460 gene loci on chromosome 1 of *Deinococcus radiodurans*, which lacks *tamA* homologs, is a *tamB* homolog (DR_146T) with two *tamB* motifs and a DUF490 motif. Mutation of DR_146T resulted in cell envelope peeling and a decrease in resistance to shear stress and osmotic pressure, as well as an increase in oxidative stress resistance, consistent with the phenotype of a surface layer (S-layer) protein SlpA (DR_2577) mutant, demonstrating the involvement of DR_146T in maintenance of cell envelope integrity. The 123 kDa SlpA was absent and only its fragments were present in the cell envelope of DR_146T mutant, suggesting that DR_146T might be involved in maintenance of the S-layer. A mutant lacking the DUF490 motif displayed only a slight alteration in phenotype compared with the wild type, suggesting DUF490 is less important than *tamB* motif for the function of DR_146T. These findings enhance our understanding of the properties of the multilayered envelope in extremophilic *D. radiodurans*, as well as the diversity and functions of TAMs in bacteria.

All Gram-negative and some Gram-positive bacteria are encased by two layers of membrane, with the outer membrane (OM) acting as a hydrophobic barrier against environmental stresses to maintain internal homeostasis and facilitate cell division^1^,^2^. The outer leaflet of many bacterial OMs is covered by a two-dimensional array of proteinaceous surface layer (S-layer)^3^. In Gram-positive bacteria and archaea, S-layers adhere to the peptidoglycan or pseudomurein, while in Gram-negative bacteria, S-layers are attached to the lipopolysaccharide (LPS) of the OM^3^,^4^,^5^. The S-layer functions as a protective barrier, but it also traps ions and is involved in cell fission^3^,^4^,^6^,^7^. S-layer proteins are secretory proteins with an N-terminal signal peptide^3^,^4^. Generally, secretory proteins are synthesized in the cytoplasm and transported through the cytoplasmic membrane by the Sec translocon system^2^,^8^,^9^, after which the signal peptide is cleaved by signal peptidase, and further transportation of the mature protein is achieved by outer membrane transportation systems including the β-barrel assembly machinery (BAM), the two-partner secretion system (TPSS) or the translocation and assembly module (TAM)^1^. The TAM consists of two components, TamA and TamB, that form a recently identified protein complex^10^ that is crucial for the assembly of outer membrane proteins^10^,^11^,^12^,^13^,^14^, as well as the virulence and colonization of pathogenic bacteria, but is not essential for viability in organisms studied thus far^10^,^14^,^15^. TamA belongs to the Omp85 protein family^1^,^10^,^15^ originally identified in *Neisseria meningitidis*^2^. TamB is an evolutionarily ancient and essential subunit of TAM^10^,^11^,^15^. Interestingly, the distribution of TamB is much broader than that of TamA, and TAM systems without TamA are found in some bacteria^15^,^16^. The properties and functions of TamB in cell envelope assembly and integrity remain to be determined.

*D. radiodurans* serves as an ideal model for studying the cell envelope and stress resistance since its initial isolation from γ-ray-sterilized canned meat^17^. To date, more than 50 species of the *Deinococcus* genus have been identified in a variety of environments^18^. *Deinococcus* are distinguished by their extraordinary tolerance to a number of lethal agents including ionizing radiation, hydrogen peroxide, osmotic pressure, desiccation, UV radiation and mitomycin C (MMC)^19^,^20^,^21^,^22^. It is widely accepted that efficient DNA repair systems and cell defence systems including an unusual cell envelope and small molecule antioxidants (e.g. Mn^2+^ and carotenoids) contribute to the survival of *D. radiodurans* under various stresses^20^,^23^,^24^,^25^,^26^,^27^,^28^. However, the mechanisms underpinning the extreme resistance of *D. radiodurans* have never been fully explained. *D. radiodurans* has an unusual multilayered cell envelope that includes a thick peptidoglycan cell wall, an outer membrane-like lipid layer and a S-layer, and it reacts positively with Gram stain despite sharing some characteristics with Gram-negative bacteria^24^,^29^,^30^. The SlpA (DR_2577) is the pivotal component of the S-layer of *D. radiodurans*. Knockout of SlpA results in the dissociation of the S-layer core structure and consequently leads to cell envelope damage^28^,^31^,^32^. Acosta and colleagues reported that SlpA of *Thermus thermophilus*, a bacterium evolutionally close to *D. radiodurans*, was assembled with assistance from the BAM complex^33^. However, the factors controlling S-layer proteins and cell envelope integrity are far from understood.

Herein, we identified a *tamB* (DR_146T) with two *tamB* motifs, even though a *tamA* homolog is not present in the genome of *D. radiodurans*^15^. Gene mutation, survival assays and cell envelope proteome analysis were performed to investigate the roles of DR_146T in cell envelope integrity with respect to SlpA and stress resistance.

## Results

### Identification of a *tamB*-containing locus in *D. radiodurans*

Two neighbouring sequences (DR_1462 and DR_1461) each containing a *tamB* motif were identified in the *D. radiodurans* genome using BLASTP, with each sequence sharing 28% and 29% amino acid sequence identity with *Escherichia coli (E. coli)* TamB encoded by *b4221*, respectively. However, a TamA homolog was not detected in *D. radiodurans* using the *E. coli* TamA as the query sequence, suggesting *D. radiodurans* might express a specific TAM lacking TamA.

**Figure 1:**
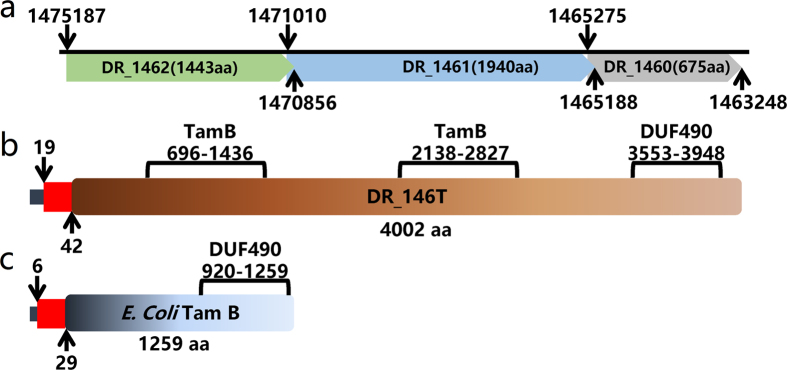
Schematic representation of *D. radiodurans* DR_146T and *E. coli* TamB. (**a**) The predicted DR_1462, DR_1461 and DR_1460 are annotated as found in the NCBI database. (**b**) The DR_146T containing two TamB motifs and one DUF490 motif was obtained from re-sequencing. (**c**) *E. coli* TamB encoded by *b4221*. Both DR_146T and *E. coli* TamB contain an N-terminal signal peptide (dark blue rectangle), which overlaps with the transmembrane segment (red rectangle).

The current *D. radiodurans* genome sequence (NCBI accession: NC_001263.1) contains three consecutive annotated ORFs (DR_1462, DR_1461 and DR_1460) in the negative chain of *D. radiodurans* chromosome 1 (Fig. 1a). By re-sequencing these loci, we found five gaps and four base errors in the DNA sequence between 1463248 and 1475251 (Supplementary Fig. S1). The gaps introduced invalid start and/or stop codons in the predicted DR_1462, DR_1461 and DR_1460 genes (Supplementary Figs S1 and S2). Therefore, DR_1462, DR_1461 and DR_1460 actually form a single intact ORF, which we refer to as DR_146T (data submitted to NCBI, GenBank accession number is KY352801). This ORF was predicted to encode a TamB homolog of 4002 amino acid residues containing two TamB domains (PFAM signature: PF04357.11) and a C-terminal DUF490 domain, on contrast with *E. coli* TamB that has one TamB domain and one DUF490 domain (Fig. 1b and c). A signal peptide of 34 amino acids and a transmembrane helix spanning residues 20–42 were predicted using the SignalIP 3.0 Server and the TMHMM Server v. 2.0, respectively (Fig. 1b). Moreover, homologs of DR_146T are also present as intact hypothetical genes in other sequenced *Deinococcus* bacteria (Supplementary Table S3). Together these features imply that the DR_146T is a *tamB* homolog^15^.

### DR_146T is involved in cell growth in *D. radiodurans*

**Figure 2:**
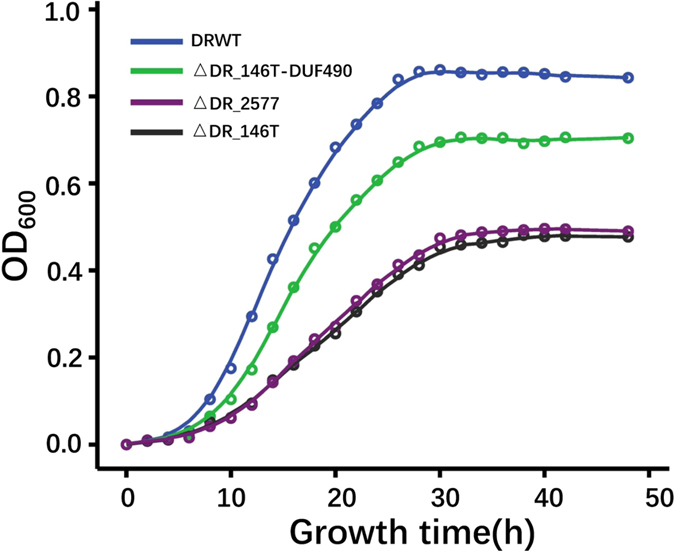
Growth of *D. radiodurans* wild type and mutant strains. The growth of DR_146T mutant (∆DR_146T, black) and *SlpA* mutant (∆DR_2577, purple) were much slower than that of the wild type (DRWT, blue), while the growth of the mutant deficient in the DUF490 motif of DR_146T (∆DR_146T-DUF490, green) was only slightly slower than that of the wild type. Cell growth was monitored by measuring the OD_600_ of cell cultures.

The growth of the DR_146T mutant (∆DR_146T) and the mutant deficient in DUF490 (∆DR_146T-DUF490) were slower than that of the wild type (Fig. 2). The DR_146T mutant grew twice as slowly as the wild type, while ∆DR_146T-DUF490 had only a slightly longer doubling time than the wild type. These results indicate that DR_146T might play an important role in cell growth or cell division. Moreover, the growth defect phenotype of ∆DR_146T is similar to that of the *SlpA* (DR_2577) mutant (Fig. 2), suggesting that DR_146T might contribute to cell envelope integrity along with the S-layer components.

### DR_146T is important for cell envelope integrity in *D. radiodurans*

**Figure 3:**
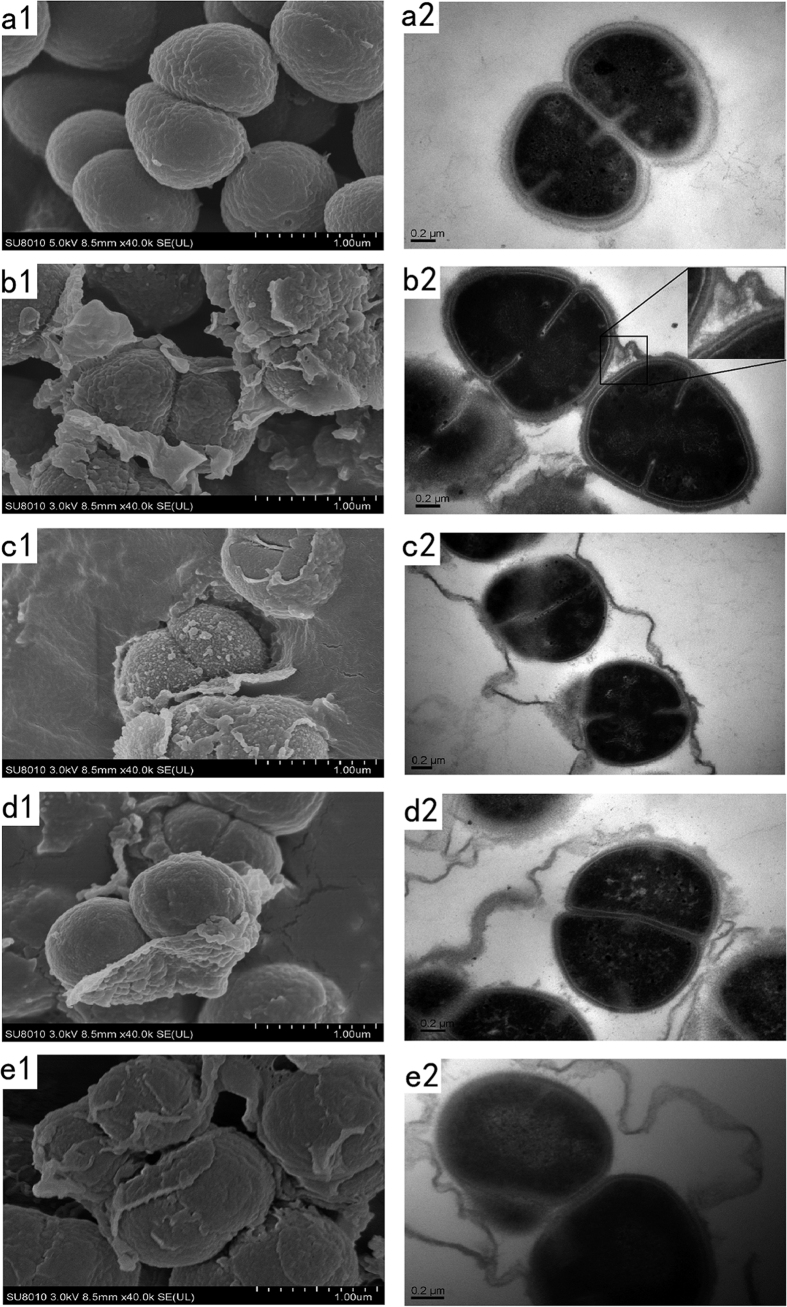
SEM and TEM images of *D. radiodurans* wild type and mutant strains. The cell envelope of ∆DR_146T exhibited severe damage with parts of the outer layer peeling off, and the inner layer exposed to the environment compared with that of the wild type. Images represent the SEM and TEM results for *D. radiodurans* wild type (a1–a2), ∆DR_146T-DUF490 (b1–b2), ∆DR_146T-TamB_2nd_ -DUF490 (c1–c2), ∆DR_146T (d1–d2) and ∆DR_2577 (e1–e2), respectively. The inset diagram in (b2) shows an amplified region of the cell envelope. Scale bars indicate the corresponding length.

The wild type colonies were circular and smooth, while the colonies of DR_146T mutant were ring-shaped and rugose similar to those of *SlpA* mutant (Supplementary Fig. S3). In liquid media, mutant cells tended to aggregate and settle more easily than wild type cells (Supplementary Fig. S4). Scanning electron microscopy (SEM) images showed that wild type cells were elliptical and grouped into diplococci or tetracocci (Fig. 3a1), whereas mutant cells displayed surface variation and shedding of the outer layer (Fig. 3b1–e1). Meanwhile, the results of transmission electron microscopy (TEM) demonstrated that the ultrastructure of the cell envelope of the mutants was different from that of wild type cells (Fig. 3a2–e2). The cell envelope of ∆DR_146T exhibited the most severe damage with parts of the outer layer peeling off, and the inner layer exposed to the environment (Fig. 3d). Damage to the cell envelope of ∆DR_146T-TamB_2nd_-DUF490 that is mutated in the second TamB and DUF490 motifs (Fig. 3c) was similar to that of the ∆DR_146T cells (Fig. 3d), but more severe than that of ∆DR_146T-DUF490 cells (Fig. 3b), indicating that TamB motif appeared to be more important than the DUF490 motif. SEM and TEM images revealed that the outmost layer peeled in the SlpA mutant (∆DR_2577) (Fig. 3e). Together with the cell growth phenotype, these results indicated that DR_146T might play an important role in maintaining cell envelope integrity. The cytoplasmic membrane (inner membrane) and peptidoglycan layer could still be formed during cell division, but formation of the outer layers was clearly inhibited in the mutant.

### Mutation of DR_146T alters stress resistance in *D. radiodurans*

**Figure 4:**
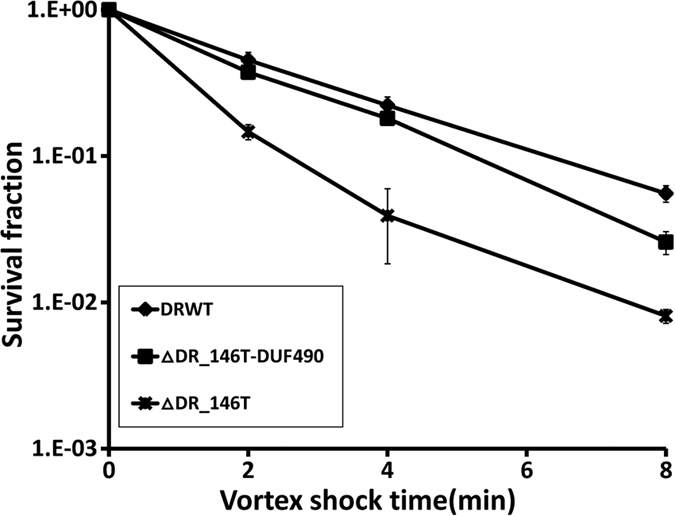
Survival of *D. radiodurans* wild type and mutant strains to shear stress. The survival fraction of ∆DR_146T was substantially lower than that for the wild type and ∆DR_146T-DUF490 under shear stress. DRWT, *D. radiodurans* wild type; ∆DR_146T-DUF490, mutant deficient in the DUF490 motif of DR_146T; ∆DR_146T, mutant deficient in DR_146T.

**Figure 5:**
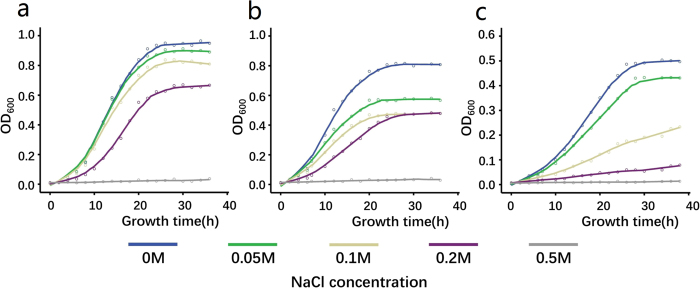
Growth of *D. radiodurans* wild type and mutant strains in TGY broth supplemented with different concentrations of NaCl. The mutants ∆DR_146T and ∆DR_146T-DUF490 were more sensitive to osmotic pressure than the wild type. (**a**) DRWT, *D. radiodurans* wild type; (**b**) ∆DR_146T-DUF490, mutant deficient in the DUF490 motif of DR_146T; (**c**) ∆DR_146T, mutant deficient in DR_146T. Growth of the bacteria was monitored by measuring the OD_600_ of cell cultures.

Under continuous vortexing, the survival fraction of ∆DR_146T was substantially lower than that of the wild type (Fig. 4), consistent with the survival phenotype of the SlpA mutant^28^. The survival of ∆DR_146T-DUF490 was slightly lower than that of the wild type. Fig. 5 shows that the cell resistance of ∆DR_146T and ∆DR_146T-DUF490 to osmotic pressure was lower than that of the wild type, indicating that mutation-induced deficiency in cell envelope integrity led to higher sensitivity to osmotic pressure. These suggest that DR_146T contributes to stress resistance, and the DUF490 motif might be less important than the TamB motif for the function of DR_146T.

**Figure 6:**
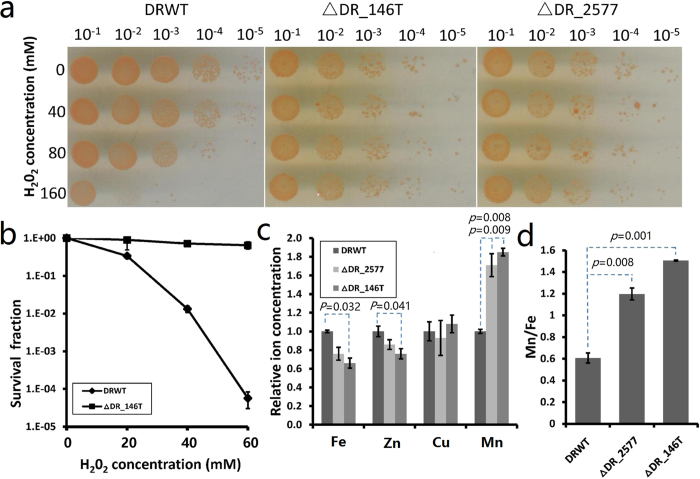
Survival of *D. radiodurans* wild type and mutant strains exposed to H_2_O_2_ treatment. The ∆DR_146T and ∆DR_2577 showed higher resistance to H_2_O_2_ and increased Mn/Fe ratio than *D. radiodurans* wild type. (**a**) Comparison of the sensitivity of wild-type (DRWT), ∆DR_146T and ∆DR_2577 strains under H_2_O_2_ treatment. Cells (10^7^ CFU ml^−1^) were dripped onto TGY plates following H_2_O_2_ treatment for 30 min and dilution with sterile phosphate buffer (0.1 M, pH 7.4). Different dilutions of cell cultures are indicated in the figure. (**b**) Survival assays of *D. radiodurans* wild type and ∆DR_146T strains. Cells were suspended in phosphate buffer (10^7^ CFU ml^−1^), treated with H_2_O_2_ for 30 min, and the survival fraction was measured by counting bacterial colonies of treated compared with the untreated samples (0 mM H_2_O_2_). (**c**) ICP-MS analysis of the relative metal ion (Fe, Zn, Cu, Mn) content in wild type and mutant strains. (**d**) Ratio of Mn and Fe ion content in wild type and mutant strains. Experiments were independently performed three times. *P-*values indicate the significance compared with wild type cells.

The resistance of ∆DR_146T and the SlpA mutant (∆DR_2577) to oxidative stress was much higher than that of wild type cells (Fig. 6a and b). The ∆DR_146T strain could survive high concentrations of H_2_O_2_ (160 mM) without any obvious decline in viability compared with controls (0 mM H_2_O_2_), indicating that disruption of DR_146T leads to an increase in resistance to oxidative stress. Previous studies demonstrated that accumulation of manganese ions (Mn^2+^) and a higher intracellular Mn/Fe ratio contribute to oxidative stress resistance in *D. radiodurans* through Mn complex-mediated ROS scavenging^25^,^26^,^34^. Thus, we measured the metal ion content in mutant and wild type cells by inductively-coupled plasma-mass spectrometry (ICP-MS) (Fig. 6c and d). The Mn ion content in ∆DR_146T and ∆DR_2577 was almost twice that of wild type cells, while the Fe and Zn ion content were slightly reduced in the mutants. Therefore, the H_2_O_2_ resistance of ∆DR_146T might be attributed to the increased Mn/Fe ratio in the mutant cells.

### Altered proteins in cell envelope, peeling fraction and culture’s supernatant

Each of the obtained whole cell envelope, culture’s supernatant and peeling cell envelope fractions was divided in half: half was used for mass spectrometry (MS) analysis, and the other half was for SDS-PAGE analysis on protein patterns of the wild type and the mutants. More than 80 proteins including DR_146T (matched to the predicted DR_1462, DR_1461 and DR_1460 from the NCBI database) were detected in the cell envelope of *D. radiodurans* wild type using MS analysis (Supplementary Table S4). Furthermore, the detection of peptides matched to the DR_146T from the cell envelope of *D. radiodurans* wild type cells by MS (Supplementary Fig. S5) suggests that DR_146T is a component of the cell envelope.

**Figure 7:**
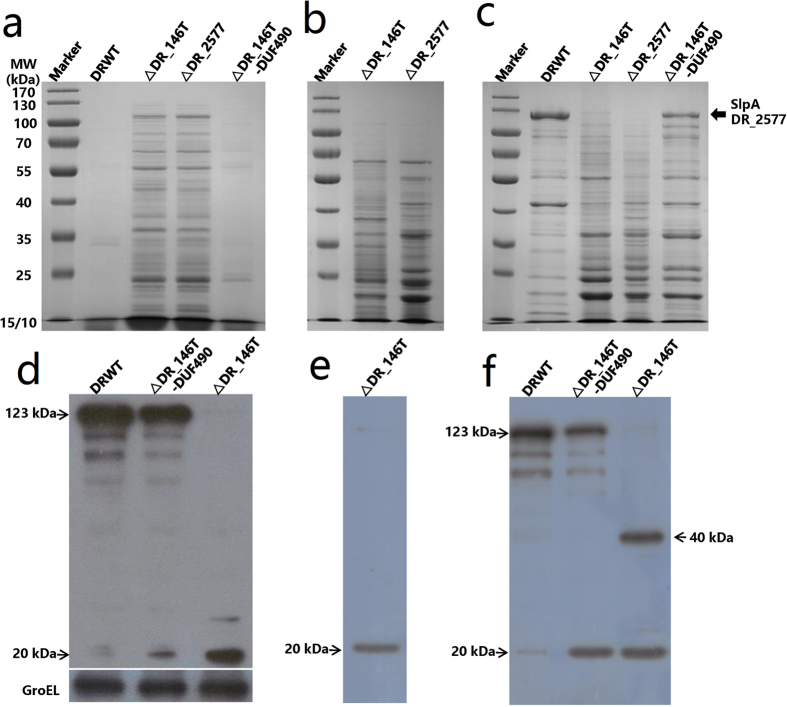
SDS-PAGE analysis of proteins (**a–c**) and western blotting of SlpA (**d–f**) in the wild type and the mutants. Compared with wild type cells, the 123 kDa SlpA (indicated by an arrow) was absent from the cell envelope and only its fragments were present in the peeling envelope fraction and whole cell envelope of ∆DR_146T. (**a**) Cell culture’s supernatant; (**b**) peeling cell envelope; (**c**) whole cell envelope separated from bacterial high pressure homogenate. (**d**) Whole cell extract; (**e**) peeling cell envelope of DR_146T; (**f**) whole cell envelope separated from bacterial high pressure homogenate. DRWT, *D. radiodurans* wild type; ∆DR_146T, mutant deficient in DR_146T; ∆DR_2577, mutant deficient in DR_2577; ∆DR_146T-DUF490, mutant deficient in the DUF490 motif of DR_146T. GroEL was used as a loading control.

SDS-PAGE results revealed some extra protein bands in the culture’s supernatant of ∆DR_146T and ∆DR_2577, however, fewer proteins were detected in the culture’s supernatant of wild type and ∆DR_146T-DUF490 cells (Fig. 7a). The protein band containing SlpA with a molecular weight of 123 kDa (Fig. 7c) was subjected to MS analysis (Supplementary Table S5). Compared with wild type cells, many proteins including the 123 kDa SlpA were absent from the cell envelope fraction of ∆DR_146T (Fig. 7b and c), suggesting that DR_146T might be involved in maintenance of the surface layer. However, the cell envelope protein profile in ∆DR_146T-DUF490 was similar to that of the wild type, confirming that DUF490 may be not a pivotal factor in maintenance of the cell surface layer.

To further probe the roles of DR_146T in cell envelope assembly and integrity, proteins in the peeling cell envelope and culture’s supernatant were identified by MS, respectively. More than 30 proteins were identified in the culture’s supernatant of ∆DR_146T (Table 1), while only a few extracellular nucleases and proteases were detected in the culture’s supernatant of wild type cells (data not shown). Among these, surface layer proteins including Hpi (DR_2508), putative S-layer-like array-related protein (DR_1115, DR_1185, DR_0383 and DR_1124) and putative periplasm-located proteins (DR_1571, DR_1027, DR_0363 and DR_A0210) were detected in the culture’s supernatant of ∆DR_146T, suggesting some cell envelope proteins were released into the supernatant following mutation of DR_146T. More than 50 proteins were identified in the peeling envelope of ∆DR_146T (Table 2), and a number of proteins detected in the culture’s supernatant were also found in the peeling envelope fraction. However, some proteins were exclusive to the peeling envelope fraction, including SlpA, the putative outer membrane protein BamA (DR_0379) and the secretin (DR_0774)^31^. SlpA was one of the most abundant proteins in the peeling fraction but was absent in the culture’s supernatant, possibly due to the presence of its SLH domain which keeps it anchored to the outer membrane^32^,^35^. Since the 123 kDa SlpA was not detected in the cell envelope of ∆DR_146T (Fig. 7c) and proteins identified by MS were based on peptide matches following enzyme digestion, the SlpA detected in the peeling fraction could be fragments of SlpA. These results indicated that many periplasmic, outer membrane and S-layer proteins or their fragments dissociated from cells and were released into the culture in the absence of DR_146T. Proper assembly of the S-layer might be inhibited by mutation of this special TamB homolog in *D. radiodurans*.

### Relationship between DR_146T and S-layer protein SlpA

SDS-PAGE revealed that a protein band of 123 kDa corresponding to SlpA was missing from the envelope of ∆DR_146T (Fig. 7c). The expression level and location of SlpA were investigated using western blotting. The 123 kDa SlpA was detected in whole-cell extracts and cell envelope fractions of the wild type and the ∆DR_146T-DUF490 cells (Fig. 7d and f). By contrast, SlpA was present only as 20 kDa and/or 40 kDa fragments in the peeling fraction and whole cell envelope of ∆DR_146T (Fig. 7e and f).

## Discussion

We identified the gene DR_146T as a *tamB* homolog in the extremophilic bacterium *D. radiodurans*, which is known for its multilayered cell envelope and exceptional stress resistance. Detection of homologs of DR_146T in other *Deinococcus* species indicates that the gene is conserved in this genus.

Evidence from several lines suggests that DR_146T is an intact functional gene. Firstly, we re-sequenced the gene loci containing the putative *tamB* homologs and found five gaps and four base errors that led to a frame shift and introduced invalid start and/or stop codons in the predicted DR_1462, DR_1461 and DR_1460 sequences. Analysis of the corrected sequences confirmed the presence of an intact ORF. Secondly, homologs of DR_146T are also present as intact ORFs in other sequenced *Deinococcus* bacteria. Thirdly, and conclusively, peptides of DR_146T were detected in the cell envelope of *D. radiodurans* wild type cells by MS analysis (Supplementary Fig. S5).

**Figure 8:**
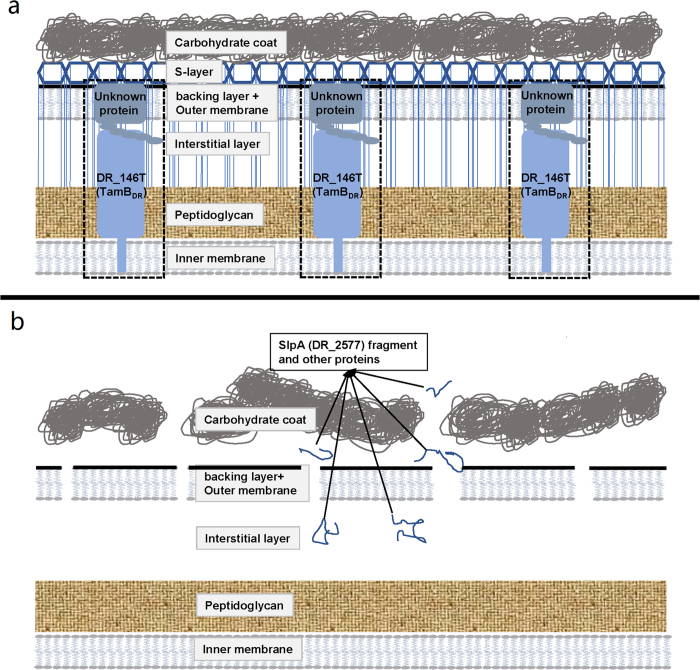
Proposed schematic diagram of the *D. radiodurans*cell envelope, adapted from previous reports^11^,^28^,^31^,^36^,^37^. (**a**) Wild type strain. (**b**) DR_146T knockout strain. The TAM complex containing the DR_146T and an unknown cooperating protein was indicated by dashed box. In wild type cells, the S-layer assembles properly and acts as a scaffold for the cell envelope. In the DR_146T mutant, SlpA (DR_2577) is present as fragments in the peeling cell envelope. Components in the carbohydrate coat, outer membrane and periplasm dissociate from the cell.

A schematic diagram of the multilayered cell envelope of *D. radiodurans* is proposed in Fig. 8, adapted from previous reports^28^,^31^,^36^,^37^. DR_146T might function as a TamB-like protein, anchored in the inner membrane with its N-terminal transmembrane helix and spanning the peptidoglycan and periplasm, based on the topology of TamB in *E. coli*^11^. However, the unusual DR_146T containing two TamB domains is larger than typical bacterial TamB which contains only a single TamB domain^15^, such as TamB in *E. coli, Citrobacter rodentium* and *Salmonella enterica*^10^. TamB in the inner membrane is reported to cooperate with TamA in the outer membrane to form a hetero-oligomeric TAM complex^10^,^11^. However, there does not appear to be a TamA homolog in *D. radiodurans*, indicating that DR_146T might act as a solo TamB. The presence of TamB in other bacteria lacking TamA has been reported, and the TAM system is suggested to have evolved from an original combination of TamB and BamA, which evolved into TamA in Proteobacteria by a later gene duplication event^15^,^29^,^38^. Whether there was any interaction between TamB and BamA in *D. radiodurans* is not clear. We did not rule out the possibility that other genes with no or little homology to *tamA* might have similar functions to *tamA* in *D. radiodurans*. Considering that the thickness of the cell envelope of *D. radiodurans* is at least twice that of *E. coli*^39^, the two TamB domains might be needed for DR_146T to span the thicker peptidoglycan and periplasm in *D. radiodurans*.

In DR_146T mutant cells, the cell envelope was disrupted and some periplasmic and S-layer proteins were released, suggesting DR_146T is involved in maintaining the integrity of the cell envelope. A number of periplasmic and S-layer proteins were detected at altered relative abundance in the peeling cell envelope of DR_146T mutants, indicating that the proteome integrity of the cell envelope was altered by the mutation. Recently, Smith *et al*. reported that the abundance of 12 proteins, including protein quality control systems and protein secretion, were altered in the membrane of the TamB homolog (MorC) mutant strain of *Aggregatibacter actinomycetemcomitans* compared with the wild type strain^40^. The main functions of the S-layer are maintaining cell shape, acting as a mechanical barrier^3^,^4^,^7^, providing a binding scaffold for large molecules and ions^41^, and mediating bacterial adhesion^42^,^43^. DR_146T might facilitate the assembly of the S-layer, because its mutation results in the loss of the 123 kDa SlpA, which is involved in formation and attachment of the surface layer to the inner cell envelope^28^. In addition, the S-layer functions as a sheath surrounding groups of cells, and forming on the surface of daughter cells when they separate^44^. In the absence of DR_146T, detachment of the S-layer sheath from the cell surface was observed in SEM and TEM images (Fig. 3). The S-layer protein of *Thermus thermophilus,* an ancient bacterial lineage closely related to *Deinococcus,* was assembled under the assistance of the BAM complex^33^. The S-layer of *D. radiodurans* may be assembled under the assistance of TamB encoded by DR_146T.

We demonstrated that deficiencies in the cell envelope following mutation of DR_146T led to increased sensitivity to shear stress and osmotic pressure due to a decrease in rigidity of the cell envelope, consistent with the SlpA mutant. Meanwhile, DR_146T and SlpA mutants showed increased resistance to oxidative stress, as well as an increased intracellular Mn^2+^ content and Mn/Fe ion ratio, both of which contribute to oxidative stress resistance through non-enzymic ROS scavenging^25^,^26^,^45^. An increase in the permeability of the cell envelope in the mutant strains might facilitate the penetration of Mn ions across the cell wall. Furthermore, we cannot eliminate the possibility that mutation of DR_146T affected metal ion transporters directly. Indeed, an iron ABC transporter substrate-binding protein (DR_B0125) was detected in the peeling cell envelope of the DR_146T mutant, suggesting that it was detached, and this could explain the observed decrease in Fe ion content in the mutants. The mechanism by which DR_146T is involved in intracellular Mn accumulation remains to be explained by further studies.

In conclusion, *tamB* homolog DR_146T in *D. radiodurans* consists of two *tamB* motifs and one DUF490 motif. DR_146T might be involved in the maintenance of cell envelope integrity and stress resistance through its impact on SlpA. Further investigations are necessary to elucidate protein conformation, structure-activity relationships and details of the mechanism of this unusual TamB homolog in cell envelope assembly. Given the important roles of the TamB homolog in cell envelope integrity, these findings are not only useful for understanding the mechanism of TAM in this and other organisms, but also valuable for screening new antibiotics that target TamB.

## Methods

### Bacterial strains and growth conditions

All bacterial strains, plasmids, and primers used in this study are listed in Supplementary Tables S1 and S2, respectively. *D. radiodurans* was cultured in TGY liquid medium (0.5% tryptone, 0.3% yeast extract, 0.1% D-glucose) or on TGY plates (TGY liquid medium supplemented with 1% agar) at 30 °C. *E. coli* was grown in LB broth (1% tryptone, 0.5% yeast extract, 1% NaCl) or on LB plates (LB liquid medium supplemented with 1% agar) at 37 °C. Appropriate antibiotics were added into the medium where required.

### Sequencing analysis of gene loci containing *tamB* motifs

Gene loci on chromosome 1 (NC_001263.1) containing *tamB* sequences were searched using BLAST (NCBI, http://blast.ncbi.nlm.nih.gov/Blast.cgi) with *tamB* from *E. coli* as the query sequence. Target DNA was sequenced using a 3730xl DNA Analyzer (ABI, USA). ORFs were predicted using OFR finder (https://www.ncbi.nlm.nih.gov/orffinder/). DNA and protein sequence analyses were performed using BLAST. Signal peptide analysis was conducted using the SignalIP 3.0 Server (http://www.cbs.dtu.dk/services/SignalP-3.0/). Transmembrane helices were analysed using the TMHMM Server v. 2.0 (http://www.cbs.dtu.dk/services/TMHMM-2.0/).

### Construction of mutant strains

Tripartite ligation and double-crossover recombination methods were used as described previously^46^,^47^ with some modifications. Briefly, DNA fragments upstream and downstream of the targeted sequence were amplified and digested with *BamH*I and *Hind*III, respectively. The streptomycin resistance fragment was amplified from vector pMD18-T and digested with *BamH*I and *Hind*III. Upstream, downstream and streptomycin resistance fragments were ligated with T4 DNA ligase, and ligation products were transformed into competent *D. radiodurans* cells using the CaCl_2_ method. The homozygous mutant strain was confirmed by PCR and DNA sequencing.

### Growth curves of mutant and wild type strains

Wild type and mutant strains were cultivated to an OD_600_ of ~1.0, inoculated into fresh TGY medium at a ratio of 1:50 (v/v) and incubated at 30 °C. Bacterial growth was monitored by measuring the OD_600_ value using a SpectraMax M5 microplate reader (Molecular Devices, USA). Experiments were performed independently in triplicate.

### Scanning electron microscopy (SEM) and transmission electron microscopy (TEM) analyses

All experimental bacterial strains were cultured to an OD_600_ of ~1.0. For SEM analysis, harvested cells were double fixed and dehydrated as described by Li^48^, and dehydrated cells were coated with gold-palladium and observed using a SU8010 ultra-high resolution SEM (Hitachi, Japan).

For TEM analysis, specimens were processed as described by Li^48^, cut into ultrathin (70–90 nm) sections using microtome, stained with uranyl acetate and alkaline lead citrate for 15 min, and observed using a Hitachi H-7650 TEM (Hitachi, Japan).

### Survival assays under shear stress, osmotic pressure and oxidative stress

Bacterial survival assays under shear stress were performed as described previously^28^ with some modifications. Briefly, bacterial cultures at an OD_600_ of ~1.0 were diluted with fresh TGY medium to OD_600_ = 0.1, mixed with sterile zirconia ceramic beads, and exposed to shear stress using a tissue homogenizer (RETSCH MM301, Germany) at 30 Hz for different treatment time (0, 2, 4 or 8 min). Suspensions were diluted and spread onto TGY plates. Bacterial colonies were counted and survival fraction was measured. For the osmotic pressure experiment, bacterial cultures at an OD_600_ of ~1.0 were inoculated into fresh TGY medium to which NaCl (0.05 M, 0.1 M, 0.2 M and 0.5 M) was added. Cultures were grown with shaking at 30 °C and the OD_600_ was measured using a SpectraMax M5 microplate reader. For the oxidative stress experiment, bacteria were treated with H_2_O_2_ in sterile phosphate buffer (0.01 M, pH 7.4) for 30 min, diluted and spread onto TGY plates. For the dripping test, 6 μl of cells was dripped onto TGY plates. All experiments were independently performed in triplicate.

### Measurement of intracellular metal ion concentration by inductively-coupled plasma-mass spectrometry (ICP-MS)

ICP-MS assays were performed as described previously by Sun^34^ with some modifications. *D. radiodurans* wild type and mutant strains were cultured to an OD_600_ of ~1.0. Cells were collected and washed three times with 2 mM EDTA in 0.01 M phosphate buffer (pH 7.4), rinsed three times with phosphate buffer without EDTA, dried and treated with nitric acid (1 M) for further metal ion concentration assays using ICP-MS (ELAN DRC-e, PerkinElmer, USA).

### Separation of cell envelope, peeling and culture’s supernatant fractions

Whole cell envelope fractions were prepared according to previously described methods^32^ with some modifications. Bacterial cultures at an OD_600_ of ~1.0 were centrifuged at 2000 g for 10 min. Cells were washed and suspended in 0.01 M phosphate buffer (pH 7.4), then disrupted at 4 °C using an ultra-high pressure continuous flow cell disrupter (JN-3000 PLUS, JNBIO, China). Undisrupted cells were removed by centrifugation (2000 g for 10 min). The supernatant was centrifuged again (20000 g for 20 min), and the envelope precipitate was washed three times in 0.01 M phosphate buffer (pH 7.4).

For separation of culture’s supernatant and peeling cell envelope fractions, bacterial cultures at an OD_600_ of ~1.0 were centrifuged at 2000 g for 10 min. The obtained supernatant was centrifuged again at 20000 g for 20 min to separate peeling and supernatant fractions. Supernatant fractions were concentrated by vacuum evaporation. Peeling fractions were washed three times with 0.01 M phosphate buffer (pH 7.4). During sample preparation, protease inhibitor cocktail (Selleckchem, USA) was added to protect proteins against proteolytic degradation.

Each of the obtained whole cell envelope, culture’s supernatant and peeling cell envelope fractions was divided in half: half was used for MS analysis, and the other half was for SDS-PAGE analyses on protein patterns of the wild type and the mutants. For electrophoresis, 6–10% (w/v) separating gels and 5% (w/v) stacking gels were used. The gels were stained with Coomassie Brilliant Blue G250.

### Identification of proteins by MS

Protein samples in solution were denatured by RapiGest SF (Waters, USA), reduced with TRIS-(2-carboxyethyl)-phosphine and alkylated with 20 mM iodoacetamide for 45 min at room temperature in darkness. Trypsin was added at 1:50 trypsin-to-protein mass ratio for digestion overnight and 1:100 trypsin-to-protein mass ratio for a second 4 h-digestion. The peptides were then desalted and dried by vacuum evaporation. For proteins in selected gel slice, the gel was diced into small pieces and reduced with TRIS-(2-carboxyethyl)-phosphine, then alkylated with 20 mM iodoacetamide for 45 min at room temperature in darkness. The obtained samples were placed into 0.65 mL siliconized tubes, then washed with 100 μl 50% acetonitrile/25 mM NH_4_HCO_3_ for 3 times and dried by vacuum evaporation. After overnight trypsin digestion, 5 μl of 5% formic acid was added to the sample to stop the reaction. The digested solution was transferred into a clean 0.65 ml siliconized tube. 50 μl 50% acetonitrile/5% formic acid was added to wash the peptides for 3 times, and the peptide supernatant was dried by vacuum evaporation. Then the peptides were diluted with 0.1% formic acid.

The peptides were analysed by HPLC-MS/MS (Triple TOF 5600 + LC/MS/MS system, AB SCIEX, USA) as described previously^28^,^31^ with some modifications. Samples were loaded on a prepacked column (200 μm × 500 mm, Chrome XP C18-CL, 3 μm, 300 Å) to desalinate for 10 min at a flow rate of 4 μl/min. Subsequently, they were eluted at 0.3 μl/min with a C18 HPLC reversed-phase column (75 μm × 150 mm, Chrome XP C18-CL, 3 μm, 300 Å) using a mobile phase of solution A (5% acetonitrile and 0.1% formic acid) and solution B (95% acetonitrile and 0.1% formic acid). During elution, solution B was increased linearly from 5% to 40% over 60 min. Eluted peptides were introduced directly into the mass spectrometer and peptide identification was performed using the *D. radiodurans* protein sequence database (NCBI). The unweighted spectrum count of each protein was divided by its mass, and the resulting index was used as an indicator of relative protein abundance in each sample^31^.

### Western blotting

Protein expression levels were confirmed using western blotting as described previously^49^. A 6× His tag was fused to the C-terminal of SlpA (DR_2577), and monoclonal anti-6× His mouse antibody (Protein tech, USA) was used to detect SlpA-6× His. Horseradish peroxidase-conjugated goat anti-mouse and anti-rabbit IgG were added as secondary antibodies. The expression level of GroEL served as an internal loading control, and was detected using rabbit anti-GroEL polyclonal antibody (Sigma, USA).

### Statistical analysis

Data were processed using SPSS 18.0 statistical software (SPSS, USA) and are presented as means ± SD. *F-*tests and independent sample *T*-tests were used to assess the significance of differences between results. *P* < 0.05 was considered statistically significant.

## Additional Information

**How to cite this article:** Yu, J. *et al*. A *tamB* homolog is involved in maintenance of cell envelope integrity and stress resistance of *Deinococcus radiodurans. Sci. Rep.* **7**, 45929; doi: 10.1038/srep45929 (2017).

**Publisher's note:** Springer Nature remains neutral with regard to jurisdictional claims in published maps and institutional affiliations.

## Supplementary Material

Supplementary Information

## Figures and Tables

**Table 1:** Proteins identified in the culture’s supernatant of ∆DR_146T by MS.

Gene Number	Protein Description	Relative Abundance Index^a^
**DR_1115**	**S-layer-like array-like protein**	++
**DR_0383**	**S-layer-like array-like protein**	+
**DR_1185**	**S-layer-like array-like protein**	+
**DR_2508**	**hexagonally packed intermediate-layer surface protein**	++
**DR_1124**	**SLH family protein**	+
**DR_A0210**	**peptide ABC transporter, periplasmic peptide-binding protein**	+
**DR_0363**	**peptide ABC transporter periplasmic peptide-binding protein**	++
**DR_1027**	**amino acid ABC transporter, periplasmic amino acid-binding protein**	+++
**DR_1571**	**peptide ABC transporter periplasmic peptide-binding protein**	++++
DR_0986	extracellular solute-binding protein	++
DR_1551	carboxyl-terminal protease	+
DR_1649	immunogenic protein	+
DR_1893	cyclophilin-type peptidyl-prolyl cis-trans isomerase	++
DR_1998	catalase	+
DR_2070	membrane lipoprotein	+
DR_2095	c-type cytochrome	+
DR_2221	tellurium resistance protein TerD	+
DR_2487	cytochrome C4	++
DR_2542	cyclophilin-type peptidyl-prolyl cis-trans isomerase	+++
DR_A0255	aculeacin A acylase	+
DR_A0283	serine protease	+
DR_0115	hypothetical protein	+
DR_0459	hypothetical protein	+++
DR_0574	hypothetical protein	+
DR_0685	hypothetical protein	+
DR_0691	hypothetical protein	+
DR_0969	hypothetical protein	++
DR_0972	hypothetical protein	+++
DR_1306	hypothetical protein	+++
DR_1406	hypothetical protein	+
DR_1805	hypothetical protein	+
DR_1940	hypothetical protein	++
DR_2319	hypothetical protein	+
DR_2320	hypothetical protein	+
DR_B0037	hypothetical protein	++

^a^Relative protein abundance was indicated by the index value (i = unweighted spectrum count of each protein/mass). +, i ≤ 0.20; ++, 0.20 < i ≤ 0.40; +++, 0.40 < i ≤ 0.60; ++++, 0.60 < i ≤ 0.80; +++++, i > 0.80.

**Table 2:** Proteins identified in the peeling cell envelope of ∆DR_146T by MS.

Gene Number	Protein Description	Relative Abundance Index^a^
**DR_2577**	**S-layer protein SlpA**	**+++**
**DR_1185**	**S-layer-like array-like protein**	**++++**
**DR_1115**	**S-layer-like array-like protein**	**+++**
**DR_0383**	**S-layer-like array-like protein**	**+++**
**DR_2508**	**hexagonally packed intermediate-layer surface protein**	**++**
**DR_1124**	**SLH family protein**	**++**
**DR_0379**	**outer membrane protein**	**+++**
**DR_0774**	**general secretion pathway protein D**	**++**
DR_0631	cell division protein FtsZ	**+**
DR_0986	extracellular solute-binding protein	+
DR_1290	extracellular solute-binding protein	+
DR_1712	extracellular solute-binding protein	+
DR_1955	extracellular solute-binding protein	+
DR_A0246	extracellular solute-binding protein	+
**DR_1571**	**peptide ABC transporter periplasmic peptide-binding protein**	++
**DR_0363**	**peptide ABC transporter periplasmic peptide-binding protein**	++
**DR_0561**	**maltose ABC transporter periplasmic maltose-binding protein**	++
**DR_1038**	**branched-chain amino acid ABC transporter periplasmic amino acid-binding protein**	++
**DR_0788**	**branched-chain amino acid ABC transporter periplasmic amino acid-binding protein**	++
**DR_0564**	**amino acid ABC transporter periplasmic amino acid-binding protein**	++
**DR_B0014**	**hemin ABC transporter, periplasmic hemin-binding protein**	++
**DR_2278**	**amino acid ABC transporter periplasmic amino acid-binding protein**	+
**DR_A0210**	**peptide ABC transporter, periplasmic peptide-binding protein**	+
**DR_1756**	**periplasmic serine protease Do**	+
**DR_0327**	**periplasmic serine protease**	+
**DR_1027**	**amino acid ABC transporter, periplasmic amino acid-binding protein**	+
**DR_1277**	**ABC transporter periplasmic substrate-binding protein**	+
**DR_2154**	**amino acid ABC transporter periplasmic amino acid-binding protein**	+
**DR_2588**	**iron ABC transporter periplasmic substrate-binding protein**	+
**DR_A0263**	**branched-chain amino acid ABC transporter periplasmic amino acid-binding protein**	+
**DR_B0125**	**iron ABC transporter substrate-binding protein**	++
DR_2070	membrane lipoprotein	++
DR_1536	serine protease	++
DR_1459	serine protease	+
DR_A0064	serine protease	+
DR_1937	serine protease	+
DR_B0007	metal binding protein	+
DR_0479	penicillin-binding protein 1	+
DR_1232	pilin, type IV	+
DR_0972	hypothetical protein	++++
DR_1021	hypothetical protein	++++
DR_2319	hypothetical protein	+++
DR_2517	hypothetical protein	+++
DR_1842	hypothetical protein	+++
DR_0116	hypothetical protein	+++
DR_1140	hypothetical protein	+++
DR_0025	hypothetical protein	++
DR_1623	hypothetical protein	++
DR_0560	hypothetical protein	++
DR_1388	hypothetical protein	++
DR_0486	hypothetical protein	++
DR_0581	hypothetical protein	++
DR_1818	hypothetical protein	++

^a^Relative protein abundance was indicated by the index value (i = unweighted spectrum count of each protein/mass). +, i ≤ 0.20; ++, 0.20 < i ≤ 0.40; +++, 0.40 < i ≤ 0.60; ++++, 0.60 < i ≤ 0.80; +++++, i > 0.80.
