# Supplementary material for: A tamB homolog is involved in maintenance of cell envelope integrity and stress resistance of Deinococcus radiodurans
Source: Sci Rep. 2017 Apr 6;7:45929. doi: 10.1038/srep45929 (PMC5382914; doi:10.1038/srep45929)
Supplement: Supplementary Information [file srep45929-s1.pdf]

Supporting Information

A *tamB* homolog is involved in maintenance of cell envelope integrity and stress resistance of *Deinococcus radiodurans*

Jiangliu Yu, Tao Li, Shang Dai, Yulan Weng, Jiulong Li, Qinghao Li, Hong Xu*, Yuejin Hua, Bing Tian*

Key Laboratory for Nuclear-Agricultural Sciences of Chinese Ministry of Agriculture and Zhejiang Province, Institute of Nuclear-Agricultural Sciences, Zhejiang University, Hangzhou, China

Corresponding author: Bing Tian (tianbing@zju.edu.cn); Hong Xu (xuhong1685@163.com), Institute of Nuclear-Agricultural Sciences, Zhejiang University, No.268, Kaixuan Road, 310029 Hangzhou, China, Tel/Fax: +86-571-86971215

Table S1. Plasmids and strains used in the experiments.

Plasmids	Description	Source
pEASY-Blunt	*E. coli* cloning plasmid	Transgen (China)
CBP-HAT-GST	GST containing plasmid	Lab stock
pRADK	*E. coli–D. radiodurans* shuttle vector	Lab stock
pMD18-T Vector	Streptomycin resistant gene containing plasmid	Lab stock
**Strains**	**Description**	**Source**
E. coli DH5α	*E. coli* cloning strain	Transgen (China)
E. coli Trans T1	*E. coli* cloning strain	Transgen (China)
D. radiodurans R1	*Deinococcus radiodurans* wild-type, ATCC 13939	Lab stock

Table S2. Primers used in the experiments.

**Re-sequencing primers**	**Sequence**
P1-F	GTTGTGACCTGCGGCGTG
P1-R	GCGCCGCGCTCGG
P2-F	CGCCCGGCTCAACCTG
P2-R	GAGCACGGTCACGCCG
P3-F	GGAGTTGCGGGCGACC
P3-R	CGTCGGAGGTCAGATGCAGG
P4-F	ACCTGCATCTGACCTCCGAC
P4-R	GCGGTGGCTCGCACG
P5-F	CGGGCGCCACGCTG
P5-R	CGCTCAGCTCGTGCCC
P6-F	GGCACCCTGACCGGCG
P6-R	CGCTGAGGGTGCCCC
P7-F	GGCACTCAGAGCGGGGC
P7-R	GTCCCGCTCGCCCG
P8-F	GGCCAGCCTGACCCGG
P8-R	TCACGTTTTCGGCGCG
P9-F	GCTGCAACTCATTCCGTCG
P9-R	GTGGAATAGGTCGCGTCGA
P10-F	GGCACCGACGGCAACG
P10-R	ATGAACTCGTTCCAGGTGCC
**Gene knockout primers**	**Sequence**
ΔDR_146T-P1	GTTGTGACCTGCGGCGTG
ΔDR_146T-P2	TATGGATCCGAGTCCCGCCCCGGTG
ΔDR_146T-P3	TATAAGCTTGGCCAACTTCCCCGGC
ΔDR_146T-P4	GCACGATGAACTCGTTCCAGG
ΔDR_146T-P5	CGCTCTACCAGAACGAACCG
ΔDR_146T-P6	CTCTGAGCCGGAATGACCAC
ΔDUF490-P1	GCGCCGGGGCGAC
ΔDUF490-P2	TATGGATCCTCACCCGGATGCGCTC
ΔDUF490-P3	TATAAGCTTGGCCAACTTCCCCGGC
ΔDUF490-P4	GCACGATGAACTCGTTCCAGG
ΔDUF490-P5	CGCTGCCCGAGGAATACA
ΔDUF490-P6	CAAGGTTGCTGCCGAGGTT
ΔTamB-DUF490-P1	GACCTGCGGGCGGTCAT
ΔTamB-DUF490-P2	TATGGATCCTCAGGCCGCCTGCCG
ΔTamB-DUF490-P3	TATAAGCTTGTGGTCACCGGGCCG
ΔTamB-DUF490-P4	GAGCGTCCAGCCTTGCTC
ΔTamB-DUF490-P5	GCCGGACATCACCTGGAAG
ΔTamB-DUF490-P6	CGGTTGCCGTGACTTTGG
ΔDR_2577-P1	AGTCTGATCGCTCTTACCACGG
ΔDR_2577-P2	CGGGATCCTTGACGGTTTCGACGCGA
ΔDR_2577-P3	CCCAAGCTTCAACGCCCTGGTCAAGGA

ΔDR_2577-P4	ACCTTGTAGCTGATCTTGAAGGTCT
ΔDR_2577-P5	CCGTTATCGTGACCTTGGG
ΔDR_2577-P6	CGTTATTGGCGTCGGTGTAG
**Tags labeled primers**	**Sequence**
DR_2577-His-P1	ACGACGGGCGATTTCAG
DR_2577-His-P2	ATAGGATCCTTAGTGATGATGATGATGATGGAA GTTGACCTTGTAGCTGATCTTG
DR_2577-His-P3	TATAAGCTTAGCCCCTAGTTCCCGGC
DR_2577-His-P4	CGGGCTGCGGAAGTGC

Table S3. Homologous proteins of DR_146T in some sequenced *Deinococcus* bacteria

Species and gene number	Annotation	Length (aa)
D. gobiensis: DGo_CA2115	Hypothetical protein	3354
D. actinosclerus: AUC44_09250	Hypothetical protein	3273
D. soli: SY84_03150	Hypothetical protein	3256
D. puniceus: SU48_10085	Hypothetical protein	3650
D. geothermalis: Dgeo_0508	Protein of unknown function DUF490	3243
D. swuensis: QR90_10050	Hypothetical protein	3272
D. deserti: Deide_16100	Conserved hypothetical protein, precursor	3511
D. proteolyticus: Deipr_1576	Protein of unknown function DUF490	3676
D. maricopensis: Deima_1996	Protein of unknown function DUF490	3180
D. peraridilitoris: Deipe_3733	Hypothetical protein	3146

Sequence information of the homologs were obtained by BLAST searching in the NCBI database (http://www.ncbi.nlm.nih.gov) using DR_146T as the query gene. The annotated homologs in the database that showed more than 25% identity (protein sequence) are listed.

Table S4. Proteins identified in the cell envelope of *D. radiodurans* wild type by MS

Gene Number	Protein Description	Relative Abundance Index[a]
**DR_2577**	**S-layer protein**	++++
**DR_0383**	**S-layer-like array-like protein**	+++
**DR_1185**	**S-layer-like array-like protein**	+++
**DR_1115**	**S-layer-like array-like protein**	+++
**DR_2508**	**hexagonally packed intermediate-layer surface protein**	+++
**DR_1124**	**SLH family protein**	+
**DR_0379**	**outer membrane protein**	+++
DR_0989	cationic outer membrane protein OmpH	+
**DR_0630**	**cell division protein FtsA**	+
**DR_0631**	**cell division protein FtsZ**	+
**DR_0774**	**general secretion pathway protein D**	+++
**DR_1460**	**hypothetical protein**	+
**DR_1461**	**hypothetical protein**	+
**DR_1462**	**hypothetical protein**	+
DR_0986	extracellular solute-binding protein	+
DR_1290	extracellular solute-binding protein	+
DR_1712	extracellular solute-binding protein	+
DR_1955	extracellular solute-binding protein	+
DR_A0246	extracellular solute-binding protein	+
DR_0561	maltose ABC transporter periplasmic maltose-binding protein	++
DR_0564	amino acid ABC transporter periplasmic amino acid-binding protein	++
DR_0788	branched-chain amino acid ABC transporter periplasmic amino acid-binding protein	++
DR_1038	branched-chain amino acid ABC transporter periplasmic amino acid-binding protein	++
DR_B0014	hemin ABC transporter, periplasmic hemin-binding protein	++
DR_B0125	iron ABC transporter substrate-binding protein	++
DR_0363	peptide ABC transporter periplasmic peptide-binding protein	+
DR_0365	peptide ABC transporter permease	+
DR_1571	peptide ABC transporter periplasmic peptide-binding protein	+
DR_0959	peptide ABC transporter permease	+
DR_1027	amino acid ABC transporter, periplasmic amino acid-binding protein	+

DR_1277	ABC transporter periplasmic substrate-binding protein	+
DR_2154	amino acid ABC transporter periplasmic amino acid-binding protein	+
DR_2278	amino acid ABC transporter periplasmic amino acid-binding protein	+
DR_2588	iron ABC transporter periplasmic substrate-binding protein	+
DR_A0210	peptide ABC transporter, periplasmic peptide-binding protein	+
DR_A0263	branched-chain amino acid ABC transporter periplasmic amino acid-binding protein	+
DR_0695	v-type ATP synthase subunit I	++
DR_0700	v-type ATP synthase subunit A	++
DR_0697	v-type ATP synthase subunit E	++
DR_0699	v-type ATP synthase subunit F	++
DR_0701	v-type ATP synthase subunit B	+
DR_0698	v-type ATP synthase subunit C	+
DR_1536	serine protease	++
DR_1459	serine protease	+
DR_2518	serine/threonine protein kinase	+
DR_0300	serine protease Do, putative	+
DR_0327	periplasmic serine protease	+
DR_1756	periplasmic serine protease Do	+
DR_1769	serine/threonine protein kinase-like protein	+
DR_1937	serine protease	+
DR_2325	serine protease	+
DR_A0064	serine protease	+
DR_0025	HSP20 family protein	+
DR_0116	hypothetical protein	++
DR_0505	5`-nucleotidase family protein	+++
DR_0560	hypothetical protein	++++
DR_0581	hypothetical protein	+++
DR_0769	hypothetical protein	+++
DR_0960	hypothetical protein	+++
DR_0969	hypothetical protein	+++
DR_0972	hypothetical protein	+++
DR_1021	hypothetical protein	+++
DR_1114	hypothetical protein	+++
DR_1140	hypothetical protein	+++
DR_1180	hypothetical protein	++
DR_1202	hypothetical protein	++
DR_1306	hypothetical protein	++
DR_1370	hypothetical protein	++
DR_1388	hypothetical protein	++

DR_1407	hypothetical protein	++
DR_1484	hypothetical protein	++
DR_1714	hypothetical protein	++
DR_1737	signal peptidase I	+
DR_1768	hypothetical protein	++
DR_1923	hypothetical protein	++
DR_1964	general secretion pathway protein E	+
DR_2070	membrane lipoprotein	++
DR_2317	potassium channel subunit beta	+
DR_2620	cytochrome c oxidase subunit I	+
DR_A0069	cleavage and polyadenylation specificity factor-like protein	+
DR_A0241	beta lactamase-like protein	+
DR_A0302	hypothetical protein	++
DR_B0037	hypothetical protein	++
DR_B0041	hypothetical protein	++
DR_B0092	Dps family DNA-binding stress response protein	+

a, Relative protein abundance was indicated by the index value (i = unweighted spectrum count of each protein/mass). +, $i \leq 0.20$; ++, $0.20 < i \leq 0.40$; +++, $0.40 < i \leq 0.60$; ++++, $0.60 < i \leq 0.80$; +++++, $i > 0.80$.

Table S5. Proteins identified in the SDS-PAGE gel containing SlpA with a molecular weight of 123 kDa by MS[a]

Gene Number	Protein Description	Relative Abundance Index[b]
**DR_2577**	**S-layer protein SlpA**	+++
DR_2508	hexagonally packed intermediate-layer surface protein	++
DR_B0037	hypothetical protein	+
DR_0363	peptide ABC transporter periplasmic peptide-binding protein	+
DR_1124	SLH family protein	+
DR_1185	S-layer-like array-like protein	+
DR_0116	hypothetical protein	+

a, The protein band containing SlpA with a molecular weight of 123 kDa in the wild type from the SDS-PAGE experiment (Fig. 7c) was subjected to MS analysis. b, Relative protein abundance was indicated by the index value (i = unweighted spectrum count of each protein/mass). +, $i \leq 0.20$; ++, $0.20 < i \leq 0.40$; +++, $0.40 < i \leq 0.60$; ++++, $0.60 < i \leq 0.80$; +++++, $i > 0.80$.

Figure S1. DNA sequence comparison of the re-sequenced DR_146T loci with NCBI data. Five gaps and four base errors in the NCBI gene loci were shown (red box). The gaps led to invalid start and/or invalid stop codons (black dashed box) in the predicted DR_1462, DR_1461 and DR_1460 sequences. The start and stop codons of DR_146T were marked with black solid line box.

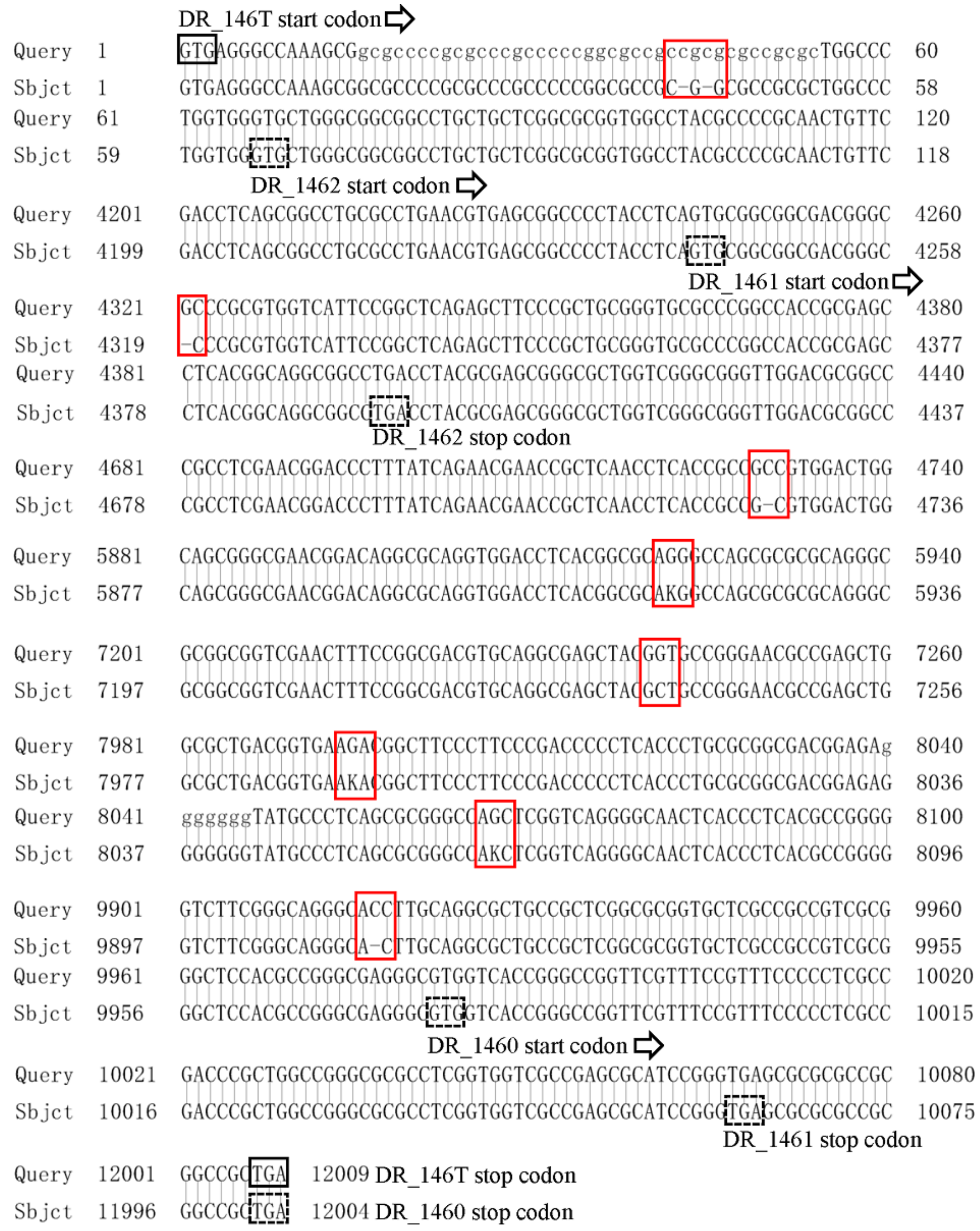

Figure S2. Predicted protein sequence encoded by DR_146T compared with the predicted protein sequences of DR_1462, DR_1461 and DR_1460 from NCBI database. Point mutation sites were indicated by red rectangle in the sequences.

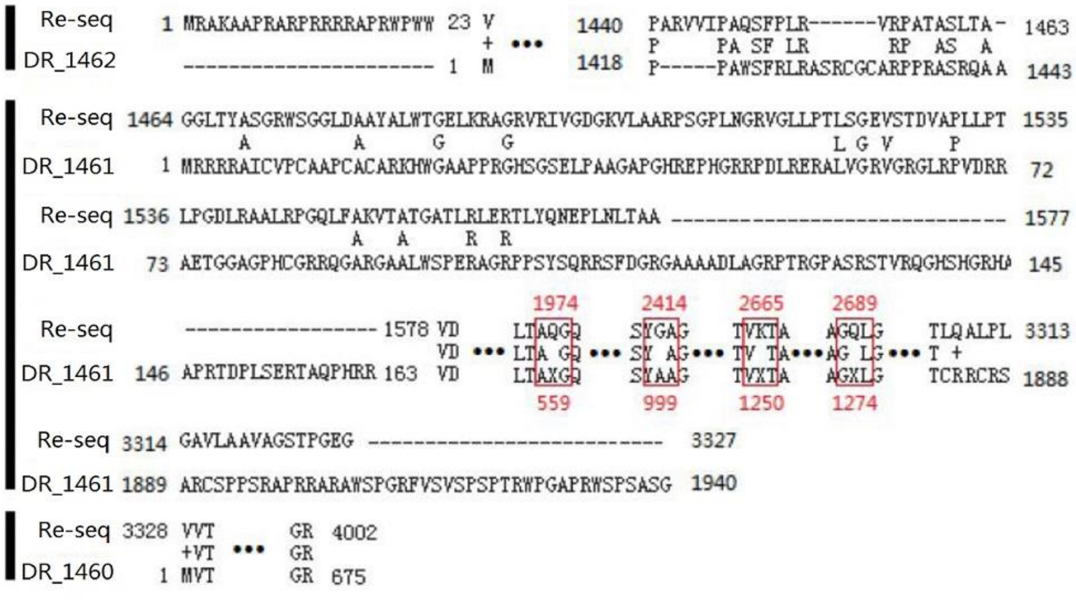

Figure S3. Comparison of colony phenotype of the wild type with those of the mutants. *D. radiodurans* wild type colonies were circular and smooth, while the colonies of DR_146T mutant were ring-shaped and rugose. DRWT, *D. radiodurans* wild type; ΔDR_146T-DUF490, mutant deficient in DUF490 of DR_146T; ΔDR_146T, mutant deficient in DR_146T; ΔDR_2577, mutant deficient in DR_2577. The inset diagram shows an amplified colony.

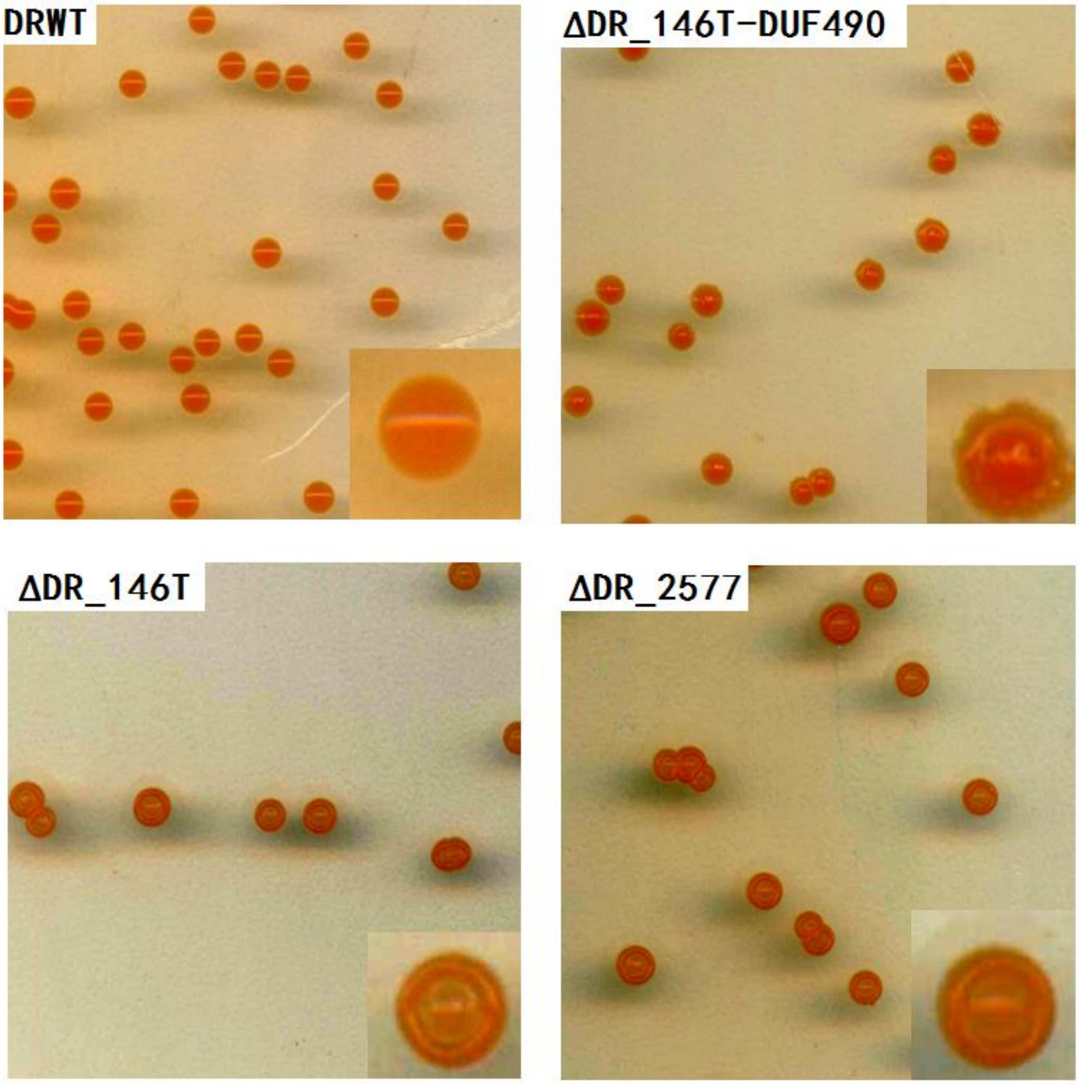

Figure S4. Settlement of bacterial cultures. *D. radiodurans* wild type and mutant cultures were placed on the table following vibration at room temperature. DRWT, *D. radiodurans* wild type; ΔDR_146T-DUF490, mutant deficient in DUF490 of DR_146T; ΔDR_146T, mutant deficient in DR_146T; ΔDR_2577, mutant deficient in DR_2577.

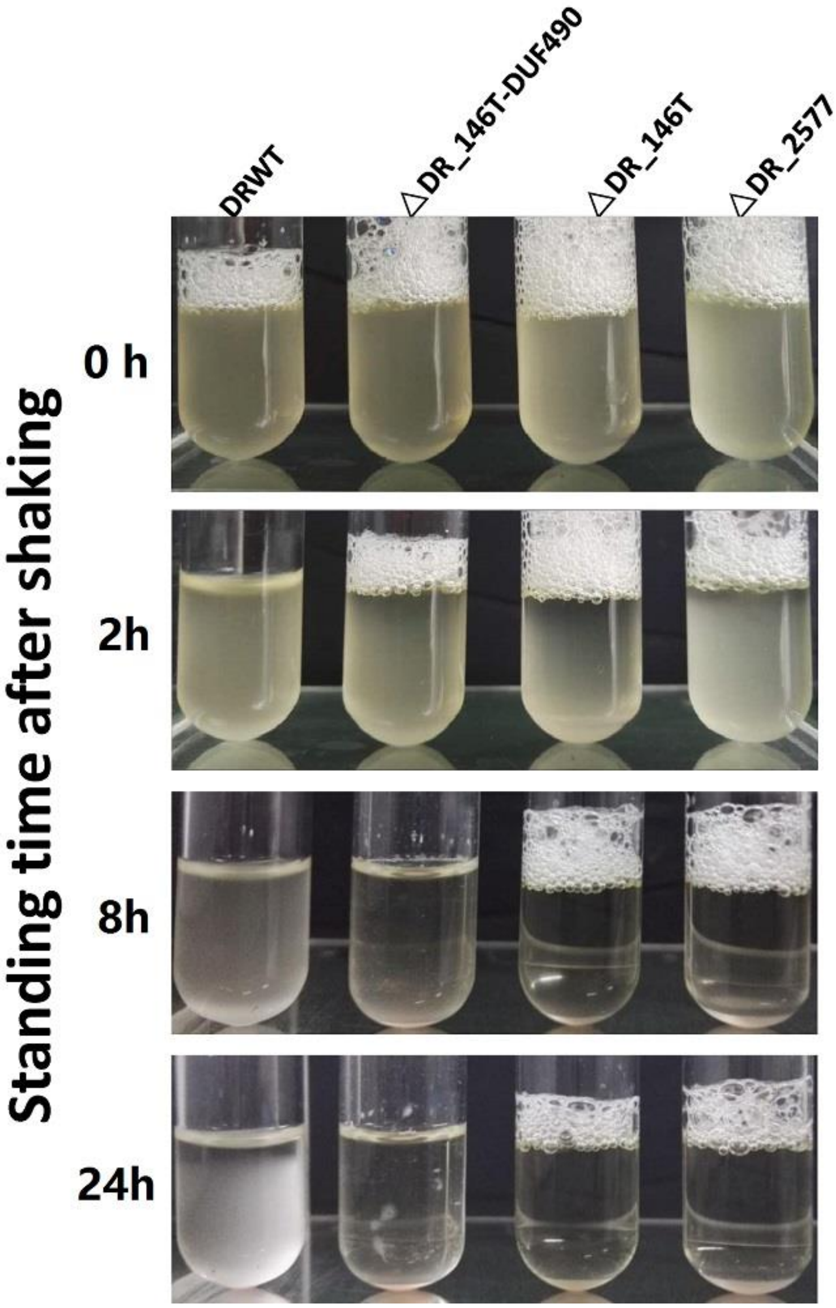

Figure S5. Peptides of DR_146T detected in the cell envelope of *D. radiodurans* wild type cells by MS analysis. The MS patterns for each peptide matched to DR_146T are shown (a-i) and the peptides are listed (j). The peptide identification was performed using the MS data obtained to search against the updated *D. radiodurans* protein sequence database supplemented with the correct DR_146T sequence.

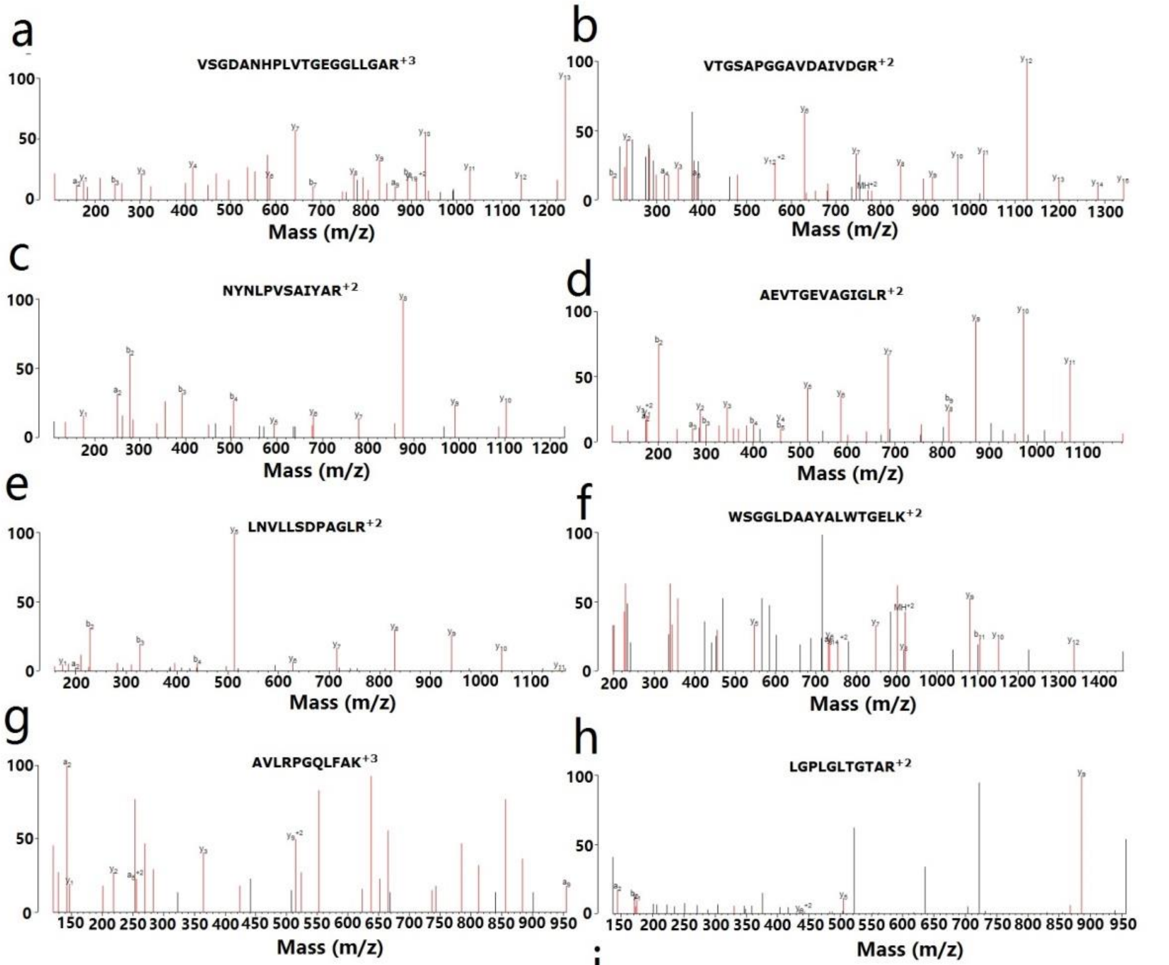

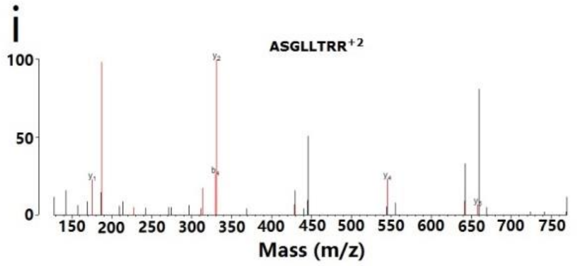

j

m/z	z	Unique peptide	RT	Score	Expect value
640.671	3	VSGDANHPLVTGEGGLLGAR	12.65	48	1.20E-10
771.399	2	VTGSAPGGAVDAIVDGR	12.917	39.7	2.10E-10
690.8635	2	NYNLPVSAIYAR	14.617	29.9	5.30E-08
636.3481	2	AEVTGEVAGIGLR	13.017	45.4	2.80E-07
634.3661	2	LNVLLSDPAGLR	15.267	34.6	1.30E-06
919.46	2	WSGGLDAAYALWTGELK	17.4	23.1	1.70E-05
400.5804	3	AVLRPGQLFAK	12.467	15.1	4.00E-04
528.2982	2	LGPLGLTGTAR	15.917	16	2.60E-03
437.2259	2	ASGLLTRR	9.517	15.2	9.50E-03
